# Editorial: theme issue on complex rheology in biological systems

**DOI:** 10.1098/rsfs.2022.0058

**Published:** 2022-10-14

**Authors:** Charley Schaefer, Gareth H. McKinley, Tom C. B. McLeish

**Affiliations:** ^1^ Department of Physics, University of York, Heslington, York YO10 5DD, UK; ^2^ Department of Mechanical Engineering, Massachusetts Institute of Technology, Cambridge, MA 01239, USA

**Keywords:** rheology, biological systems, flow, deformations

## 1. Introduction

Recent years have witnessed unprecedented growth in interdisciplinary engagement and collaboration of physical and life sciences, to which the very existence of the Royal Society Journal ‘Interface Focus’ testifies. The subject of rheology itself brings together physics, chemistry, chemical engineering, mathematics and computing. In the biological context, the interdisciplinarity becomes even richer. Cell biology in plants, animals and prokaryotes is usually described in terms of components, biochemical networks and signalling. Yet local flows, and deformations of the entire cell as well as its individual parts [1,2], are essential to function. Questions on such mechanical properties and phenomena are rarely addressed. At the tissue biology level, there are new challenges especially in the highly nonlinear range of deformations [3,4], coupling to smaller structures, and pathologies. Vascular biology (e.g. haematology) is clearly a field where rheology is vital [5], but other key rheological control problems emerge in digestive [6] and reproductive biology [7]. Other biological flows contain rheologically induced structural or phase transitions, and the understanding of how biological fluids and soft solids flow and deform is a key scientific area within this collaboration of physical and life sciences (blood [8], the cytosol, silk protein solutions [9], saliva, mucus [10], synovial fluid, biofilms [11–13], tissue buckling [14], bacterial rheotaxis [15] and *E. coli* bacteria swimming in media with liquid crystalline order [16] are just some examples [17]).

This theme issue on ‘Complex rheology in biological systems’ brings together biorheological work across distinct disciplines in the physics and life sciences. Below, we summarize these contributions and extract common ideas and methodologies. We hope this will promote their adoption across the field, and thereby accelerate the resolution of outstanding and unresolved problems in biorheology. Beyond that, this theme issue aims to raise awareness of new research questions that have not yet been fully formulated in some of the sub-fields, yet are key to a wider understanding. As is generally true in the ‘physics of living matter’ movement, the biological examples point to new physics and chemistry that is not evident in non-biological systems.

## 2. Contributed works

For this theme issue, we gratefully received contributions from across the physics and life sciences with interests in biorheology ranging in length scale from the rheological properties of intracellular biomolecular networks [1,10] to the scale of the direct extracellular environment [3,7,10,12,13,16], tissues [3–5] and even entire organs that actively exert forces onto non-Newtonian fluids [6]. The mechanical properties at the cellular level are discussed in relationship to cancer [2], as well as in relationship to the transport of red blood cells in disordered porous environments, be it in vascular networks or in microfluidic devices. The interaction of a cell with its environment, and in particular the consequence of these interactions to cell mobility, is discussed in the context of (i) blood flow in vascular networks [5], (ii) male infertility [7], (iii) the stability of biofilms [12,13] and (iv) new opportunities of using liquid crystals to impose controlled constraints to cell motion [16].

Common themes in these works involve non-Newtonian fluids and bio-molecular structures that may exhibit a non-affine passive stress–strain response, or which may actively exert a force on the fluid. While the properties of the fluids and structures are often poorly understood, the level of complexity increases still further when the fluids and structures interact. Such flow–structure interaction is important in achieving quantitative evaluation from video defaecography for diagnostics [6], and is also important for understanding how blood flows through microvessels and vascular tissues [5]. Our understanding of flow can be improved experimentally by controlling a rigid flow geometry in microfluidic devices [5]. This enables the study of the consequences of the elasticity of red blood cells to flow. The measurement of the elastic properties of living cells is highly challenging, but is achieved by Lee *et al*. by developing a monolayer rheometer [2]. To study the motion of cellular microswimmers, their ‘run-and-tumble’ behaviour can be frustrated in a controlled way using liquid crystals [16].

The challenge of understanding intra- and extracellular biomolecular networks across length scales requires identification of whether the network is passive or active [3], and the level of pre-stress [4]. To assess this information, the contributed review by Erlich *et al.* [4] argues it is necessary to develop novel non-perturbative methodologies to probe the network at a small length scale. Lecinski *et al.* address this using single-bead tracking passive rheology in live *S. cerevisiae* yeast cells [1], and Jory *et al.* discuss new methodologies to probe mucus adhesion at the microscopic scale using optical tweezers [10]. Erlich and co-workers also argue the interpretation of the force–displacement measurements in such experiments relies on the development of suitable theoretical models, which face the challenge of linking the molecular topology of the network to the mechanics at the continuum level; such a modelling contribution is provided by Song and co-workers who discuss hyperelastic continuum models [3]. These activities strongly resonate with the contribution by Jory *et al.* who have studied the adhesion of mucus at the microscopic scale and shear-thinning and elasticity at the macroscale. They discuss how rheological measurements on mucus from human bronchial epithelial cultures may be used for the diagnosis of chronic obstructive pulmonary disease [10].

## 3. Discussion and conclusion

Current experimental design has increasingly moved towards a sophisticated simultaneous probe of microscopic structure and its deformation as well as macroscopic stress and strain. Examples are neutron-scattering, and IR or NMR spectroscopies in flow-fields, as well as complex flow visualization in three dimensions (e.g. via dynamic confocal microscopy). Microrheological methods exploit Brownian motion and diffusion to probe the rheological properties of complex fluids at different length scales. Theoretical models have developed towards coupling across scales, so that microscopic theory underpins the emergent flow properties of complex fluids. The physical (and engineering) field of classical bulk ‘rheology’ and the molecular and statistical physics underpinning ‘rheophysics’ have driven the development of advanced experimental techniques that operate at both microstructural and emergent macroscopic length scales, and can trace the complex viscoelasticity of fluids containing polymers, membranes and nanoparticles, to their molecular causes. At the same time, theoretical and computational tools that describe the statistical physics of soft matter have also developed, which are able to account for long-standing rheological puzzles such as the role of polymer branching in generating extensional strain hardening, and the ‘jamming’ transition of sheared particles at high density in suspension.

These tools from the physical sciences are now sufficiently mature for application to systems with the additional complexity afforded by the biological examples considered in the present issue. The fundamental structures are the same at a coarse-grained level (denatured proteins are polymers, globular proteins are effectively colloidal nanoparticles and lipid membranes are surfactant self-assemblies, at first approximation). However, the evolved specificity and detailed structural ordering in biological materials, the ubiquity of charged groups, counter-ions, hydrophobic interactions, and above all the recognition that these are active and driven systems out of equilibrium, create two great opportunities for the interdisciplinary science of rheology. The first is to deploy physical rheological methods to understand biological and biomedical examples (such as the unique rheological signatures of cancer cells and tumours). The second is in the identification, description and understanding of fundamentally new rheophysical phenomena embodied by biological systems (such as ‘active rheology’). This theme issue brings together research on such phenomena, but with a complementary focus on the key challenges when viewed through the different disciplinary lenses. It is essentially (in Royal Society language) a way to bring together ‘A-side’ and ‘B-side’ researchers who do not normally attend the same conferences. The Hooke/Theo Murphy meeting complementing this theme issue (to be held in Summer/Autumn 2023) will hopefully differ from the usual rheology conferences in attracting a good number of researchers who would not think of themselves as ‘rheologists’ but who would both benefit from, and contribute to, the evolution of the discipline. We hope that the wider consequences might be very promising and rich. In the same way that metastases in cancer were shown to have an important rheological aspect, there are other possible physical–biomedical connections that may offer new therapeutic avenues. There are also other possible insights and synergies to be learned from bioinspired materials processing. Here, the spinning of silk dopes offers an example of solution-processed fibre formation that is orders of magnitude more efficient than current artificial processes. The food and personal care industries are replete with examples of bio-formulated rheology and processing challenges. We seek to actively involve discussants and participants from across the industrial, biological and medical fields to consider the rheological and soft material science opportunities that are entwined with these key societal challenges.

## Data Availability

This article has no additional data.
